# Carbon and nitrogen allocation shifts in plants and soils along aridity and fertility gradients in grasslands of China

**DOI:** 10.1002/ece3.3245

**Published:** 2017-07-28

**Authors:** Wentao Luo, Mai‐He Li, Jordi Sardans, Xiao‐Tao Lü, Chao Wang, Josep Peñuelas, Zhengwen Wang, Xing‐Guo Han, Yong Jiang

**Affiliations:** ^1^ Erguna Forest‐Steppe Ecotone Research Station Institute of Applied Ecology Chinese Academy of Sciences Shenyang China; ^2^ Swiss Federal Research Institute WSL Birmensdorf Switzerland; ^3^ CSIC Global Ecology Unit CREAF‐CEAB‐UAB Cerdanyola del Vallès Catalonia Spain; ^4^ CREAF Cerdanyola del Vallès Catalonia Spain; ^5^ State Key Laboratory of Vegetation and Environmental Change Institute of Botany Chinese Academy of Sciences Beijing China

**Keywords:** biomass allocation, climatic gradient, nutrient availability, plant chemistry, transect

## Abstract

Plant carbon (C) and nitrogen (N) stoichiometry play an important role in the maintenance of ecosystem structure and function. To decipher the influence of changing environment on plant C and N stoichiometry at the subcontinental scale, we studied the shoot and root C and N stoichiometry in two widely distributed and dominant genera along a 2,200‐km climatic gradient in China's grasslands. Relationships between C and N concentrations and soil climatic variables factors were studied. In contrast to previous theory, plant C concentration and C:N ratios in both shoots and roots increased with increasing soil fertility and decreased with increasing aridity. Relative N allocation shifted from soils to plants and from roots to shoots with increasing aridity. Changes in the C:N ratio were associated with changes in N concentration. Dynamics of plant C concentration and C:N ratios were mainly caused by biomass reallocation and a nutrient dilution effect in the plant‐soil system. Our results suggest that the shifted allocation of C and N to different ecosystem compartments under a changing environment may change the overall use of these elements by the plant‐soil system.

## 1 INTRODUCTION

Plant carbon (C) and nitrogen (N) stoichiometry play a fundamental role in the maintenance of ecosystem structure and function (Yuan & Chen, 2015). Carbon is the substrate and energy source of physiological and biochemical processes; nitrogen is an important component of plant proteins and nucleic acids. Plant C and N stoichiometry reflects the relative strength of C and N metabolism, and can thus be used to predict plant growth and development (Elser, Fagan, Kerkhoff, Swenson, & Enquist, 2010; Luo et al., 2015). Therefore, understanding variation in plant C and N stoichiometry has been a key focus for both plant physiologists and ecologists.

Most climate change scenarios forecast that several regions will undergo increasing aridity (IPCC 2013; Meehl & Tebaldi, 2004). Soil fertility is projected to change around the globe as a result of imbalances in field fertilization, atmospheric N deposition, pollution, land use changes, and climate change among other processes (Peñuelas, Sardans, Rivas‐ubach, & Janssens, 2012). Climate and soil fertility are key drivers of ecosystem processes at the regional scale. Projected changes in environmental factors will have a large influence on plant C and N stoichiometry in natural ecosystems (Lu, Yan, Fan, Cao, & Wang, 2011; Luo et al., 2015; Sardans, Rivas‐Ubach, & Peñuelas, 2012; Yuan & Chen, 2015). Therefore, understanding the sensitivity of the relative internal concentrations of C and N in individual primary producers and in communities to global environment changes is a high priority.

Geographical gradients provide an opportunity to decipher the effects of changing environments (e.g., precipitation and soil fertility) on variation in plant C and N, which is important for understanding the underlying patterns of C and nutrient fluxes and the mechanisms of the response of vegetation to ongoing global environmental changes. Water availability in temperate ecosystems can influence the rate of plant growth and the availability of soil N, leading to changes in plant N concentration ([N]) and C:N ratios in aboveground biomass (An et al., 2005). Altered climates and soil fertility can also lead to changes in plant size (Li & Yang, 2004; Li et al., 2013), rates of plant production (Guo et al., 2012; Huxman et al., 2004), metabolism, and patterns of biomass allocation (Luo et al., 2013; Yang, Fang, Ma, Guo, & Mohammat, 2010), consequently resulting in variation in C and nutrient composition (Elser et al., 2010; Luo et al., 2015) because various plant structures and metabolic processes have distinct and divergent requirements for C and other nutrients (Elser et al., 2010). These relationships, however, are very complex and vary in their roles in the effect of the stoichiometry of plant organs on plant growth and production and the variation with plant size in nutrient allocation to different organs (Elser et al., 2010).

To decipher the influence of changing environment on plant C and N stoichiometry, we studied the shoot and root C and N stoichiometry in two widely distributed and dominant genera along a 2,200‐km terrestrial transect in China's grasslands. The selected grassland transect exhibits pronounced climate gradients and is little influenced by direct human perturbations, such as industrial and agricultural land use (Kang, Han, Zhang, & Sun, 2007; Zhang et al., 2014), which provides an ideal platform for examining the potential shifts in plant C and N stoichiometry under the projected scenarios of global environmental change (Peng et al., 2013; Zhang et al., 2014).

We hypothesized that changes would occur in the allocation of C and N in the soil‐plant system with increased aridity and between above‐ versus belowground organs within a plant. We also hypothesized that the changes would differ between C (entering ecosystems from atmosphere) and N (entering ecosystems from the atmosphere and soils) and among different species because of niche segregation among sympatric species.

## 2 MATERIALS AND METHODS

### 2.1 Study area and sampling

In early August 2012, we conducted the study along a 2,200‐km west–east transect in China's grasslands (Fig. S1). The transect spanned 105.6°E–120.4°E and 40.7°N–50.1°N. The topography of the study region consists of gently rolling hills and tablelands, with elevations ranging from 1,500 m above sea level in the west to 700 m above sea level in the east. The climate of this region is markedly seasonal, with substantial annual variation in both temperature and precipitation (Guo et al., 2012; He et al., 2006). From west to east along the transect, mean annual temperature (MAT) decreased from +7 to −2°C, and mean annual precipitation (MAP) increased from 100 to 450 mm. Across the broad geographical regions and environmental gradients along the west–east transect, there are three natural vegetation types (i.e., desert steppe, typical steppe, and meadow steppe from west to east) (Guo et al., 2012). The soil type of the region is predominantly xeric, sandy, brown loess rich in calcium classified in the Kastanozem group in the classification system of the Food and Agriculture Organization. From west to east along the transect, organic C concentration ([C]) and total [N] in the topsoil (10 cm) varied systematically; organic [C] ranged from 0.1% to 4.5%; total [N] ranged from 0.01% to 0.35%; soil C:N ratio (mass‐based) increased from five to 13. *Stipa* and *Cleistogenes* are two widely distributed and dominant plant genera in the semiarid grassland ecosystems. *Stipa* spp. are perennial multi‐stem C3 bunchgrasses and *Cleistogenes* spp. are multi‐stem C4 bunchgrasses.

A total of 37 sites were established along the transect, with approximately 60 km between sites (Fig. S1). The selected sampling sites were 500–1,000 m away from major roads and >50 km from human settlements and were subjected to minimal animal grazing and other anthropogenic disturbances. Sampling locations were GPS referenced based on latitude, longitude, and elevation (eTrex Venture, Garmin, USA). At each site, a 50 × 50‐m plot was randomly established in an area that was flat or with <1% slope, with relatively homogenous soils and vegetation cover and no obvious signs of animal disturbance. Then, five 1 × 1‐m subplots were established at the four corners and in the center of the plot. Mature and healthy individuals belonging to each genus (*Stipa* and *Cleistogenes*) were selected and collected from each subplot. The majority of root material of these plants was located in the upper 30 cm of soil. The root system was excavated by extracting a soil cylinder of approximately 15–25 cm in diameter and 20–30 cm in depth with the target plant in the middle, using a spade. The size of the soil cylinder depended on the shoot morphology of the target plant. Roots of the target plant were carefully collected and separated from soils and other root materials. Shoots and roots were separated and temporarily stored in an envelope. Furthermore, aboveground biomass of the rest of plants belonging to the two genera within each subplot was harvested by clipping at the ground level. In the laboratory, all living plants were oven‐dried and weighed to calculate the biomass allocation of each genus. Two of the 37 sampling sites along the transect had no *Stipa* spp., and nine sampling sites had no *Cleistogenes* spp. We, therefore, collected a total of 175 samples of *Stipa* spp. from 35 sampling sites and 140 samples of *Cleistogenes* spp. from 28 sampling sites (see Fig. S1 and Table S1).

In each subplot, soil samples (0–10 cm) were also collected after removing the litter layer. Ten randomly allocated soil cores were collected at each subplot using a soil auger 2.5 cm in diameter and were mixed to produce one composite sample per subplot. Roots and rocks were removed from the soil samples, which were then stored in cloth bags at room temperature for the analysis of soil chemistry. More detailed descriptions of the study region and the sampling protocol can be found in our previous publications (Luo et al., 2013; Wang et al., 2014).

### 2.2 Measurements

The plant shoot and root samples were washed with running deionized water to remove soil, and they were dried at 65°C to a constant weight. Then, shoot‐to‐root ratios were calculated as the dry shoot biomass (g) divided by the dry root biomass (g). The dried samples were ground to a fine powder using a ball mill. Plant [C] and total [N] were then determined using an elemental analyzer (2,400 II CHNS/O Elemental Analyzer, Perkin‐Elmer, USA) at a combustion temperature of 950°C and a reduction temperature of 640°C.

The soil samples were air‐dried and ground to a fine powder using a ball mill. Carbonates were removed from the samples using 0.5 M HCL following the method described by Harris, Horwáth, and van Kessel (2001). Soil organic [C] and total [N] were measured using an elemental analyzer at the Stable Isotope Facility of the University of California, Davis.

We extracted MAP and annual potential evapotranspiration (PET) from a global climate database with 1‐km resolution (http://www.worldclim.org/) (Hijmans, Cameron, Parra, Jones, & Jarvis, 2005). This climatic model was based on interpolated values of climatic data (1950–2000) provided by weather stations throughout the region and adjusted to the topography. Aridity is defined as 1‐AI, where AI, the ratio of MAP to PET, is the aridity index. A value of 1 for aridity means most arid, while a value of 0 means no aridity (because MAP = PET).

A variety of indices can be used as surrogates of soil fertility. We selected the two most commonly used indices, soil total [N] (the size of the nutrient pool) and the C:N ratio (quality of the organic matter), for analyzing the effects of soil fertility on plant [C] and C:N stoichiometry (Ordoñez et al., 2009).

### 2.3 Statistical analyses

The whole plant, shoot and root C and N contents were calculated as the product of biomass by concentration. The C and N shoot‐to‐root content ratios were calculated as the shoot C and N contents divided by root C and N contents to detect changes in the allocation of C and N to shoots versus roots in the two genera along the soil and climatic gradient.

Regression analyses between plant [C] and C:N ratios and aridity were conducted to demonstrate the effects of climatic regime on plant chemistry. We similarly performed regression analyses between plant [C] and C:N ratios and soil variables (total [N] and C:N ratios) to explore the effects of soil fertility on plant chemistry. The statistical analyses were conducted using SPSS 13.0 (SPSS Inc., Chicago, USA, 2004).

To study the relationships between aridity shifts and the overall plant, shoot, root, and soil [C] and [N] and C:N ratios, we have used structural equations modeling (SEM). The SEM analyses also allow the detection of indirect effects of aridity on these plant traits through the effects of aridity on soil [C] and [N] and C:N ratios. We also introduced into the SEM analysis the genera (as a dummy variable) as an exogenous factor that can affect the relationships between aridity and overall plant‐soil [C] and [N] and C:N ratios. Different sympatric species can produce litter with different elemental compositions, and their species‐specific litter elemental compositions can also shift differently when the aridity increases. Therefore, we also used the aridity, soil [C] and [N], and genera (as a dummy variable) as factors explaining the maximum variability in the total plant, shoot and root [C] and [N], and finally C:N ratios in the overall plant biomass, shoots, and roots. This further assessed the relationships among these variables, and the shifts in C and N fluxes among compartments and the amounts stored in each plant‐soil compartment related to increased aridity. We fitted the different models using the *sem* package in R (Fox, Nie, & Byrnes, 2013) and determined the most parsimonious model using the Akaike information criterion. Standard errors and the significance level (P) of the total, direct, and indirect effects were calculated using bootstrapping (1200 iterations).

## 3 RESULTS

### 3.1 Relationships of plant C:N stoichiometry with aridity and soil fertility

**Figure 1:**
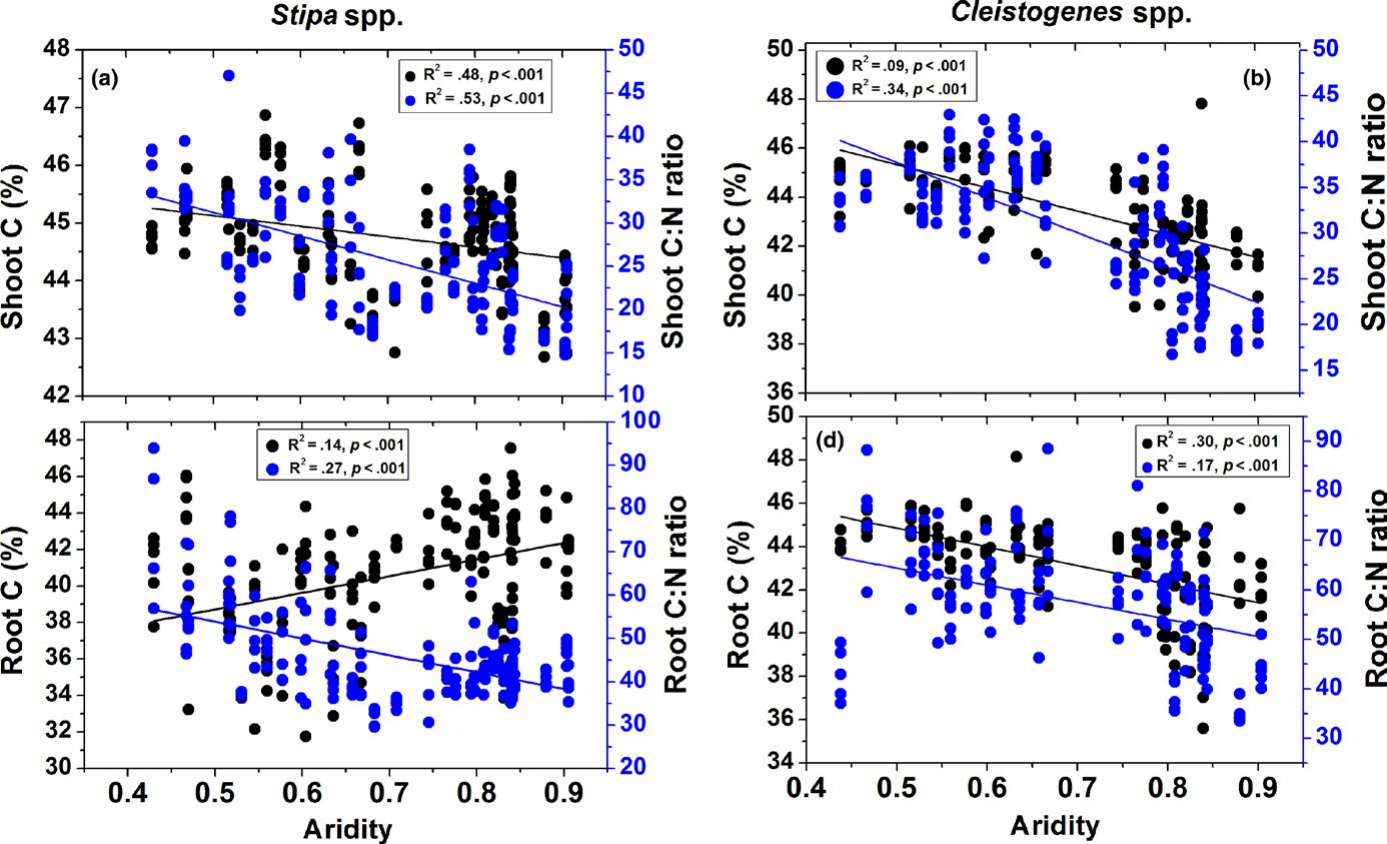
Relationships between plant shoot and root [C] and C:N ratios and aridity for *Stipa* and *Cleistogenes* in China's grasslands. Left *Y*‐axis corresponds to solid black circles; right *Y*‐axis corresponds to solid blue circles

Plant shoot [C] significantly decreased with increasing aridity for *Stipa* and *Cleistogenes* (all *p *<* *.001; Figure 1). Plant root [C] increased with increasing aridity for *Stipa* but decreased for *Cleistogenes* (all *p *<* *.001; Figure 1). Shoot and root C:N ratios decreased with increasing aridity for *Stipa* and *Cleistogenes* (all *p *<* *.001; Figure 1).

**Figure 2:**
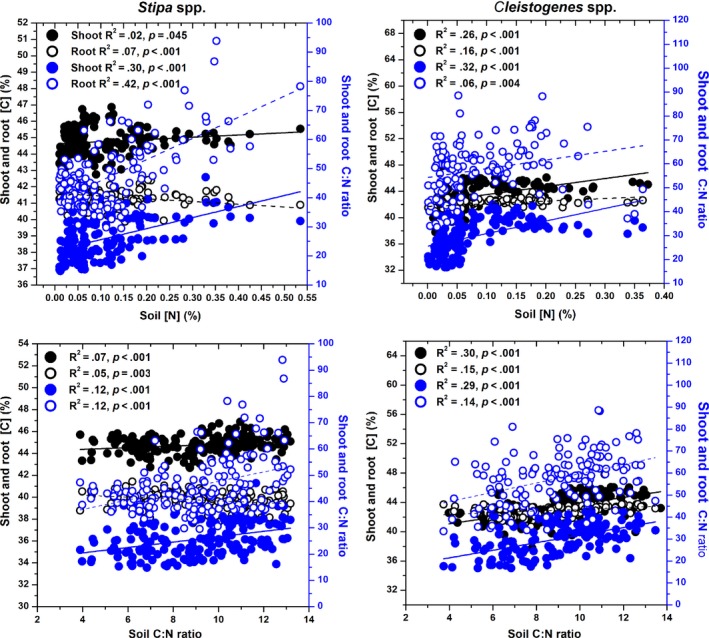
Relationships between plant shoot and root [C] and C:N ratios and soil fertility (soil total [N] and C:N ratio) for *Stipa* and *Cleistogenes* in China's grasslands. Left *Y*‐axis corresponds to open and solid black circles; right *Y*‐axis corresponds to open and solid blue circles

Shoot [C] was positively correlated with soil total [N] and soil C:N ratios for *Stipa* and *Cleistogenes* (all *p *<* *.001; Figure 2). Plant [C] in the root tissues of *Stipa* decreased linearly with soil total [N] and soil C:N ratios (all *p *<* *.01; Figure 2). The relationships between root [C] of *Cleistogenes* and soil fertility were similar to those of shoot (both *p *<* *.001; Figure 2). Plant shoot and root C:N ratios were significantly positively correlated with soil total [N] and C:N ratios for *Stipa* and *Cleistogenes* (all *p *<* *.01; Figure 2).

**Figure 3:**
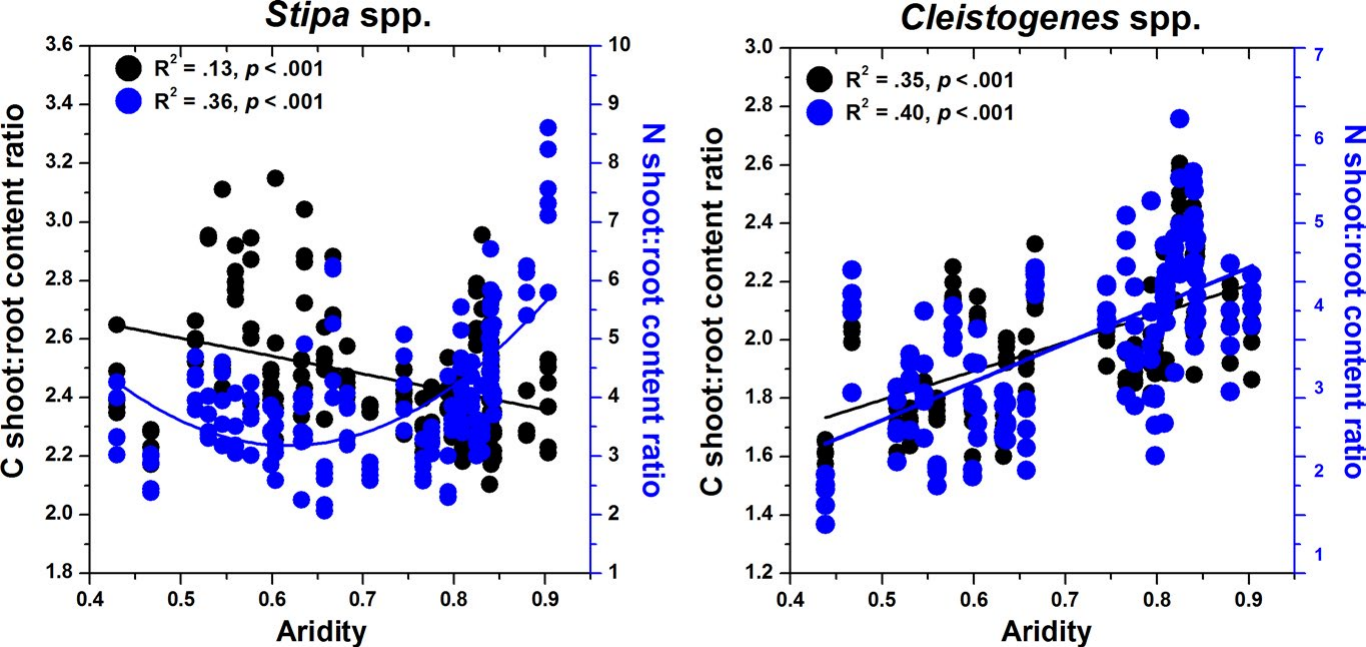
Relationships between aridity and the C and N shoot‐to‐root content ratios for *Stipa* and *Cleistogenes* in China's grasslands. Right *Y*‐axis corresponds to black circles; right *Y*‐axis corresponds to blue circles

Change in C and N shoot‐to‐root content ratios along the climatic gradient differed between the two genera, mainly for the C contents (Figure 3). The C shoot‐to‐root content ratios in *Stipa* decreased linearly along the aridity gradient, and the N shoot‐to‐root content ratios first decreased and then increased above an aridity of 0.6 (Figure 3). Both the C and N shoot‐to‐root content ratios increased in *Cleistogenes* along the aridity gradient (Figure 3).

**Figure 4:**
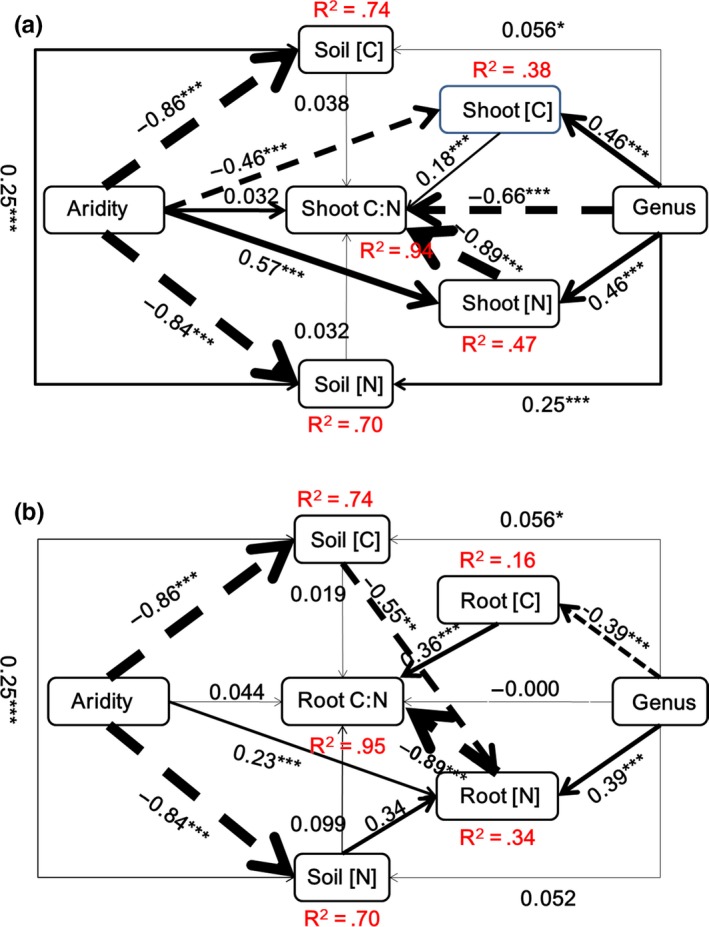
Diagrams of the structural equation models that best explained the maximum variance in the shoot (a) and root (b) C:N ratios and aridity, soil and plant [C] and [N], and genera (*Stipa* and *Cleistogenes*) as an exogenous factor. Dashed and solid arrows indicate negative and positive relationships, respectively. Numbers below them (between brackets) indicate likelihood estimates between the two corresponding variables and the corresponding level of significance (*p*‐value). *p <* *.05; **p <* *.01, ***p <* *.001. Arrow width is proportional to the strength of the relationship

**Figure 5:**
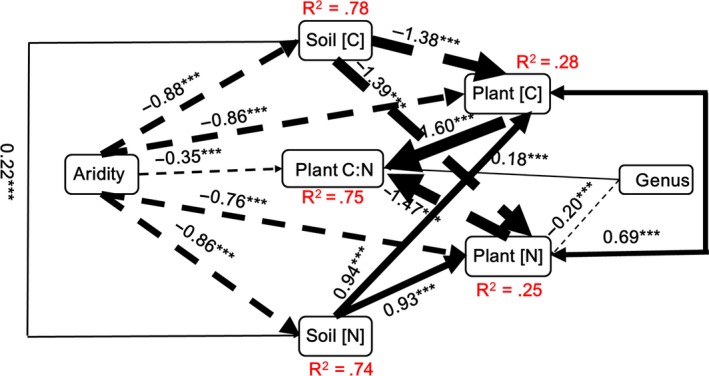
Diagrams of the structural equation models that best explained the maximum variance in the plant C and N contents (m^−2^) and aridity, soil and plant [C] and [N], and genera (*Stipa* and *Cleistogenes*) as an exogenous factor. Dashed and solid arrows indicate negative and positive relationships, respectively. Numbers below them (between brackets) indicate likelihood estimates between the two corresponding variables and the corresponding level of significance (*p*‐value). ***p <* *.001. Arrow width is proportional to the strength of the relationship

The SEM analysis showed that the effect of aridity on the shoot and root [C] and [N] was mainly due to its direct rather than an indirect effect on soil [C] and [N], despite the significant negative correlations between aridity and soil [C] and [N] (Figures 4 and 5). Moreover, the effect of aridity on the shoot and root C:N ratios was mainly due to its indirect positive correlation with shoot and root [N] and not to a direct effect or to an indirect effect on shoot and root [C] (Figures 4 and S3). Aridity had a significantly negative effect on total biomass C:N ratios, indicating a greater increase in [N] than [C] with increased aridity (Figures 5 and S4). The effect of aridity was greater on [N] than on [C] (Fig. S5).

### 3.2 Relationships between plant [C] and [N]

The large variability in plant C:N ratios in response to the climatic and soil variables was mainly driven by plant N dynamics (Fig. S2). Plant C:N ratios were more strongly associated with [N] than [C] in the aboveground tissues for *Stipa* (R^2^ = 0.932 for N vs. 0.075 for C; Fig. S2) and *Cleistogenes* (R^2^ = 0.926 for N vs. 0.414 for C; Fig. S2). Similarly, plant belowground C:N ratios were significantly negatively correlated with plant N dynamics for *Stipa* and *Cleistogenes* (both *p *<* *.001) but had no clear relationships with plant C dynamics (Fig. S2).

## 4 DISCUSSION

Plant [C] and C:N ratios increased with increasing water availability. Higher plant [C] and C:N ratios have been correlated with lower water availability (Chen, Wang, Xiong, Cao, & Deng, 2015; Sardans et al., 2012), and a relatively constant [C] per unit of dry mass has been demonstrated in plant tissues exposed to altered climatic regimes (He et al., 2006) and at elevated CO_2_ levels (Luo, Hui, & Zhang, 2006). However, our results were more in line with those from a study that also showed a decrease in [C] in plant aboveground biomass after exposure to drought stress (Alam et al., 2013) and with those from a study that reported a significantly positive relationships between [C] in fine roots and rainfall (Yuan, Chen, & Reich, 2011). Increased [C] and C:N ratios with increasing water availability may be attributed to shifts in the partitioning of biomass to biomechanical support tissues with relatively higher [C] but lower [N], such as stems and coarse roots (Aerts & Chapin, 2000; Elser et al., 2010). We previously found that precipitation stimulated the rate of plant growth and increased plant size (biomass and height) in grassland ecosystems in these drylands (Luo et al., 2013), indicating that plants need to invest and allocate more C to stems and course roots (Aerts & Chapin, 2000; Elser et al., 2010; Sterner & Elser, 2002), which may further dilute the [N] in those tissues, resulting in higher [C] and lower [N] and hence higher C:N ratios (Li et al., 2013; Luo et al., 2006).

Soil fertility plays a crucial role in regulating plant nutrients and dynamics in terrestrial ecosystems (Castle & Neff, 2009). The biogeochemical hypothesis, one of the central paradigms of ecological stoichiometry, proposes that the availability of soil nutrients related to precipitation and temperature is the main driver of plant C:nutrient stoichiometry (Reich & Oleksyn, 2004). Fertile soils can provide mineral nutrients for plant uptake and growth, so despite the large variability in plant strategies, plants growing on more fertile soils generally contain higher nutrient concentrations and thus relatively lower C:nutrient ratios (Oleksyn, Reich, Zytkowiak, Karolewski, & Tjoelker, 2003; Ordoñez et al., 2009). However, contrary to this prediction, plant [C] and C:N ratios in our study increased significantly with soil fertility, likely due to the higher accumulation of [C] than [N] associated with an increased rate of plant growth, which would increase N‐use efficiency (i.e., C:N ratio) with increasing soil fertility.

The positive correlations between plant C:N ratios and soil total N content and C:N ratio may also be partly explained by a dilution effect associated with plant size (Marba, Duarte, & Agusti, 2007). Soil fertility and water are limiting factors in the arid and semiarid grassland ecosystems in our study area, so higher availabilities of water and soil nutrients will increase plant height, biomass production (i.e., plant size), and shoot‐to‐ root ratio (Luo et al., 2013), diluting the nutrient concentrations in the plant tissues and consequently leading to an increase in plant C:nutrient ratios. These results suggested a shift in plant‐soil system shift, in terms of both growth and nutrient allocation, in the sense of having a higher ability to conserve nutrients in plant organs, alleviating their dependence on soil nutrient availability as soil infertility increased, as observed in previous studies (Drenovsky, James, & Richards, 2010).

The shifts in plant C:N ratios for both genera were largely determined by plant N dynamics. Two meta‐analyses by Yang, Luo, Lu, Schädel, and Han (2011) showed that plant C:N ratios were not correlated with corresponding plant C but were negatively correlated with plant N dynamics, which was similar to our results. These patterns in our study may have been caused by the relatively larger variations in [N] than [C] for the two genera. For example, shoot [N] in *Stipa* ranged from 1.0% to 3.0% (a 200% increase), whereas shoot [C] increased from 42.7% to 46.9% (only a 10% increase), therefore the dynamics of plant shoot C:N stoichiometry were primarily driven by N rather than C dynamics along the environmental gradients.

Different relationships between [C] and [N] were found in shoot and root tissues. These results are in line with those for global secondary forests, where C and N dynamics acted independently during stand development (Yang & Luo, 2011). The different relationships between [C] and [N], however, does not agree with the results of our previous study, which showed that C and N were biologically coupled, from cellular metabolism to ecosystem structure and nutrient cycling, by their effects on the biochemical reactions that control primary production, respiration, and decomposition in terrestrial ecosystems (Finzi et al., 2011; Schlesinger, Cole, Finzi, & Holland, 2011). The different relationships between [C] and [N] were associated with the differences in their concentrations in various plant tissues, which vary widely across broad geographical regions and environmental gradients, as discussed above. Such asymmetrical effects of climate and soil fertility on plant [C] relative to [N] in biomass would affect nutrient turnover and water‐use efficiency and consequently the capacity to sequester C (Sardans, Penuelas, & Ogaya, 2008).

The decoupling of plant [C] and [N] in above‐ and belowground plant organs with climatic shifts can also be observed at the level of plant shoot/root allocation of nutrients. A global climatic shift represented by aridity indicated that aridity was associated with lower [C] and [N] and contents in soils and total plant biomass (based on a decrease in biomass, despite the higher concentrations) and with a higher allocation to shoot tissues within the plant (except for C in *Stipa*). Our results showed a higher proportional allocation of N to shoot rather than root tissues with increasing aridity for both genera along the gradient. All these results showed a shift in C and N allocation from soil to plants and within plants from roots to shoots with increasing aridity.

In summary, this study identified the effects of climate and soil fertility on plant [C] and C:N ratios in both the above‐ and belowground tissues of two widely distributed and dominant genera on a large scale. Plant [C] increased with soil fertility but decreased with aridity mainly due to biomass reallocation and the dilution effect. Carbon and N allocation shifted from soil to plants and within plants (except C in *Stipa* spp.) from roots to shoots with increasing aridity. The C cycle was asynchronous from the N cycle in plants, and the allocation of C and N to different ecosystem compartments shifted under a changing environment, which in turn may decrease the provision of ecosystem services, shift the overall use of these elements by the plant‐soil system and have further feedbacks on climate change. These findings thus have clear implications for understanding the processes regulating C and N cycling in grasslands throughout these regions and provide important information regarding the successful introduction of the N cycle into current general C and climatic models. Our study achieved broader spatial scales but suffered from a lack of control over covariates. In the future, only integrating such approach with experimental approach can allow us to examine the comprehensive responses of ecosystem to climatic changes.

## CONFLICT OF INTEREST

None declared.

## AUTHOR CONTRIBUTIONS

W.L, M.L., Y.J., Z.W., X.L., and X.H. designed the study; W.L, X.L., and X.H. performed the experiment, and W.L, J.S, J.P., and Y.J. analyzed the data and wrote the paper. All coauthors participated in discussions during the working group meetings and edited the manuscript.
