# Supplementary material for: Carbon and nitrogen allocation shifts in plants and soils along aridity and fertility gradients in grasslands of China
Source: Ecol Evol. 2017 Jul 28;7(17):6927–34. doi: 10.1002/ece3.3245 (PMC5587465; doi:10.1002/ece3.3245)
Supplement: Supplementary file 1 [file ECE3-7-6927-s001.docx]

**Supporting information for**

**Carbon and nitrogen** **allocation shifts in plants and soils** **along aridity and fertility gradients in grasslands of China**

Wentao Luo, Mai-He Li, Jordi Sardans, Xiao-Tao Lü, Chao Wang, Josep Peñuelas, Zhengwen Wang, Xing-Guo Han, Yong Jiang

**Table S1** Mean plant chemistry ([C], [N], and C:N ratios) in plant above- and belowground tissues for *Stipa* and *Cleistogenes* along the transect.

**Figure S1** The 2200-km transect in China's grasslands. A total of 37 sites from west to east were selected along the transect.

**Figure S2** Relationships of C:N ratios with [C] and [N] for *Stipa* and *Cleistogenes* in China's grasslands.

**Figure S3.** Total, direct, and indirect effects of aridity, plant and soil [C] and [N], and genera (*Stipa* and *Cleistogenes*) on C:N ratios in shoots and roots. Data were obtained by bootstrapping (1200 repetitions). Aridity is defined as 1-AI, where AI, the ratio of potential evapotranspiration to precipitation, is the aridity index. *, *P*<0.05; **, *P*<0.01, ***, *P*<0.001.

**Figure S4.** Total, direct, and indirect effects of aridity, plant and soil [C] and [N], and genera (*Stipa* and *Cleistogenes*) on total plant biomass C:N ratio. Data were obtained by bootstrapping (1200 repetitions). Aridity is defined as 1-AI, where AI, the ratio of potential evapotranspiration to precipitation, is the aridity index. *, *P*<0.05; **, *P*<0.01, ***, *P*<0.001.

**Figure S5.** Diagrams of the structural equation models that best explained the maximum variance in the whole plant C and N concentrations and aridity, soil and plant [C] and [N], and genera (*Stipa* and *Cleistogenes*) as an exogenous factor. Numbers below them (between brackets) indicate likelihood estimates between the two corresponding variables and the corresponding level of significance (*P*-value). *, *P*<0.05; **, *P*<0.01, ***, *P*<0.001. Total, direct, and indirect effects of aridity, plant and soil [C] and [N], and genera (*Stipa* and *Cleistogenes*) on total plant biomass C:N ratio. Data were obtained by bootstrapping (1200 repetitions). Aridity is defined as 1-AI, where AI, the ratio of potential evapotranspiration to precipitation, is the aridity index.

|  | *Stipa* spp. | | | | | | *Cleistogenes* spp. | | | | | |
| --- | --- | --- | --- | --- | --- | --- | --- | --- | --- | --- | --- | --- |
|  | Aboveground | | | Belowground | | | Aboveground | | | Belowground | | |
|  | [C] | [N] | C:N | [C] | [N] | C:N | [C] | [N] | C:N | [C] | [N] | C:N |
| No. of sites | 35 | 35 | 35 | 35 | 35 | 35 | 28 | 28 | 28 | 28 | 28 | 28 |
| No. of plant samples | 175 | 175 | 175 | 175 | 175 | 175 | 140 | 140 | 140 | 140 | 140 | 140 |
| Mean (%) | 44.8 | 1.9 | 25.7 | 40.6 | 0.9 | 46 | 43.4 | 1.5 | 30.0 | 43.1 | 0.8 | 57.4 |
| Maximum (%) | 46.9 | 3.0 | 47 | 47.6 | 1.4 | 93.9 | 47.8 | 2.6 | 43.0 | 48.1 | 1.4 | 88.5 |
| Minimum (%) | 42.7 | 1.0 | 14.8 | 31.8 | 0.5 | 29.5 | 37.8 | 1.1 | 16.7 | 35.6 | 0.5 | 33.5 |

**Table S1**

**
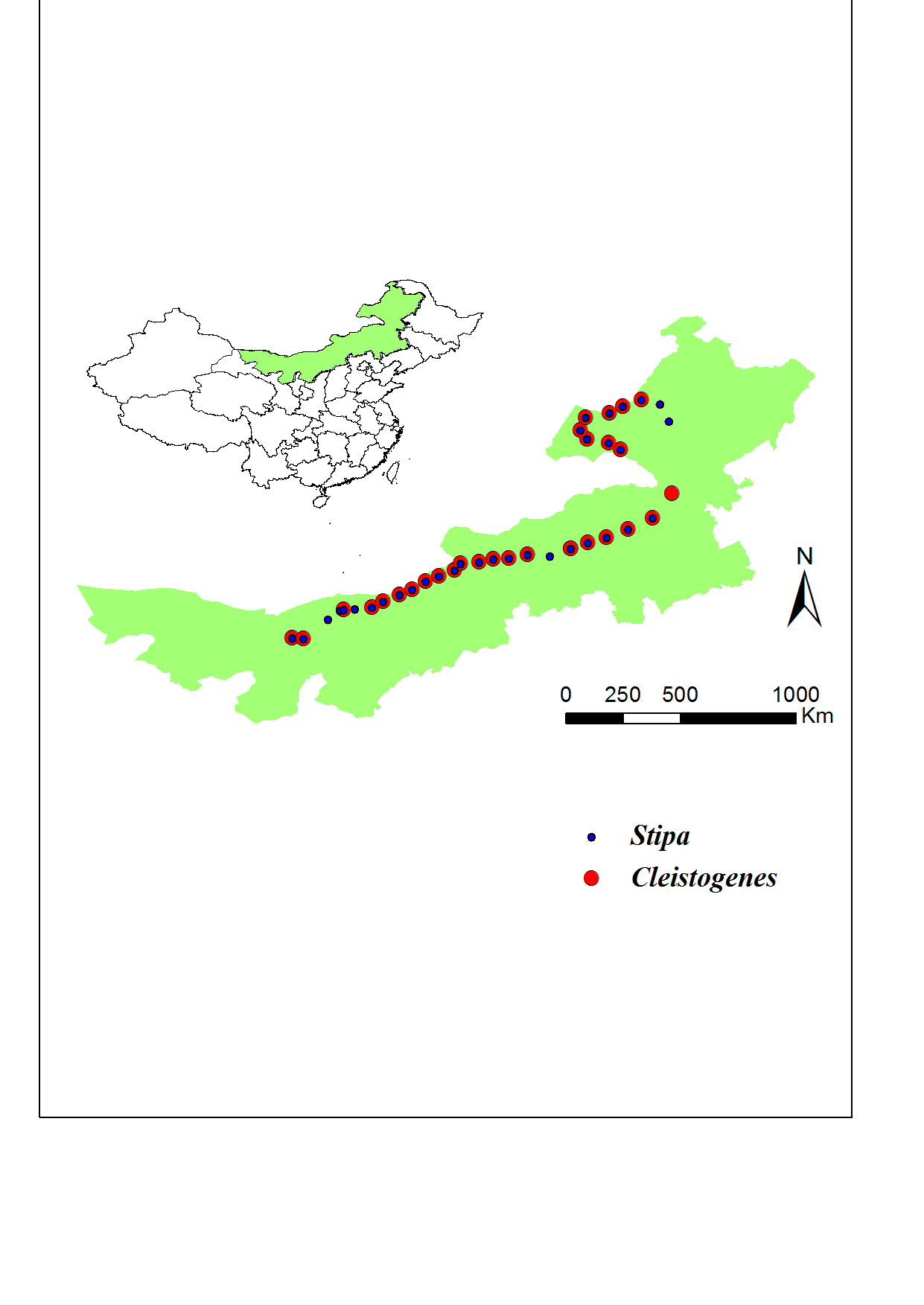
**

**Figure S1**







**Figure S2**


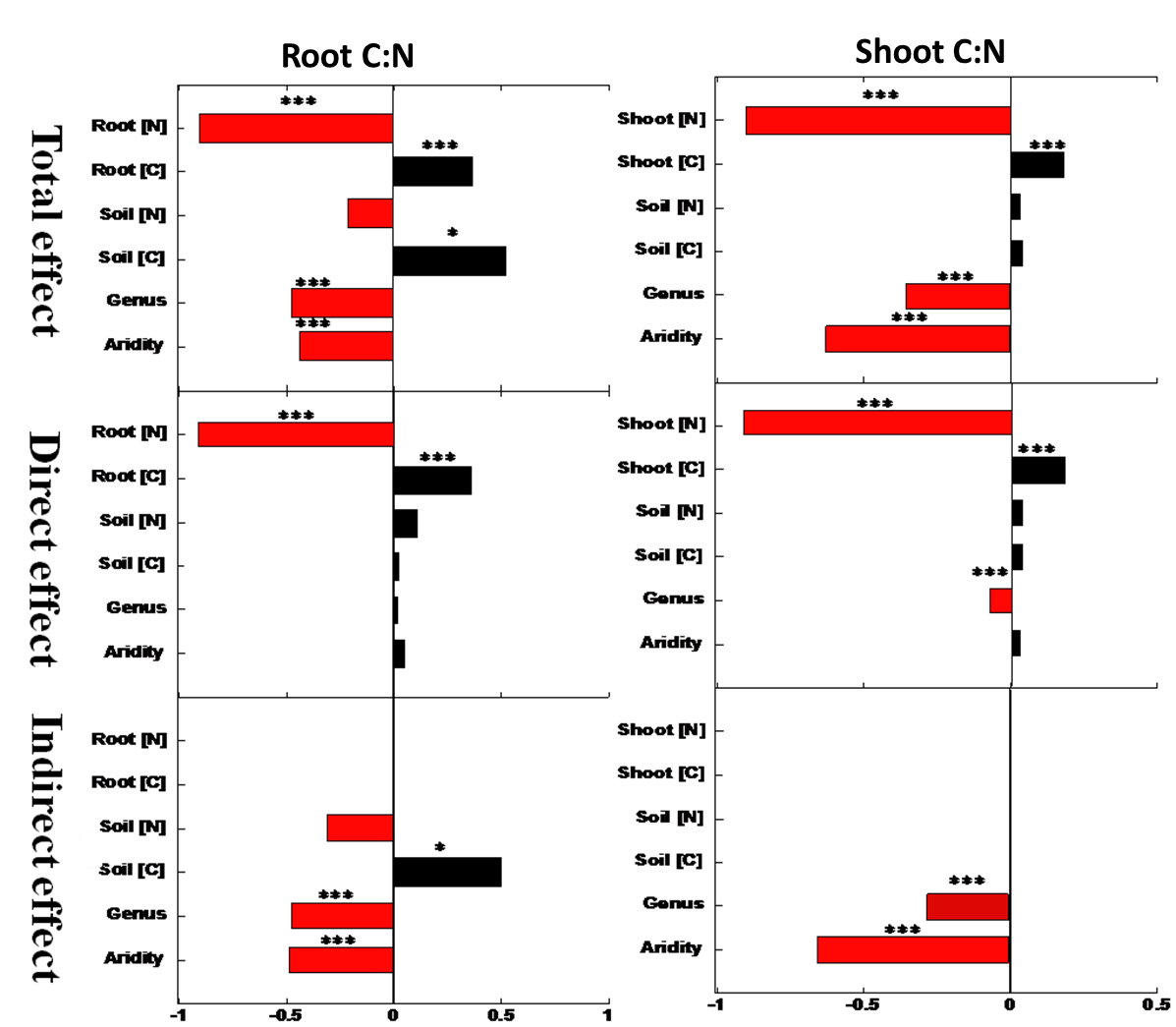


**Figure S3**

**Figure S4**

**Figure S5**
